# Community-Based Non-Pharmacological Intervention for Metabolic Syndrome in Chile: A Comparative Reanalysis of an Integrated Exercise and Health Education Program

**DOI:** 10.3390/ijerph23080955

**Published:** 2026-07-24

**Authors:** Guillermo Medina-Santos, Alberto Urzúa, Jorge Méndez-Cornejo, Edgardo Rojas-Mancilla, Carmen Zambrano-Bravo, Esteban Oñate-Henríquez, Trinidad Parada, Cristian Vidal-Silva

**Affiliations:** 1 Facultad de Salud y Servicios Sociales, Universidad Estatal de Milagro (UNEMI), Milagro 091002, Ecuador; jmedinas@unemi.edu.ec; 2 Escuela de Kinesiología, Facultad de Salud, Universidad Santo Tomás, Campus Talca, Talca 3460000, Chile; 3 Department of Physical Activity Sciences, Faculty of Education Sciences, Universidad Católica del Maule, Talca 3460000, Chile; jmendez@ucm.cl; 4 Fundación Arturo López Pérez OECI Cancer Center, Laboratorio Clínico, Santiago 7500000, Chile; edgardo.rojas@falp.org; 5 Departamento de Ciencias del Movimiento Humano, Facultad de Ciencias de la Salud, Universidad de Talca, Talca 3460000, Chile; czambrano@utalca.cl; 6 Departamento de Ergonomía, Facultad de Ciencias Biológicas, Universidad de Concepción, Concepción 4070386, Chile; estebanonate@udec.cl; 7 Servicio Local De Educación Pública Los Alamos, Linares 3580000, Chile; trinidad.parada@sleplosalamos.gob.cl; 8 Facultad de Ingeniería y Negocios, Universidad de Las Américas, Manuel Montt 948, Santiago 7500975, Chile

**Keywords:** metabolic syndrome, lifestyle intervention, aerobic exercise, health education, cardiometabolic risk, functional capacity, community health

## Abstract

**Highlights:**

**Public health relevance—How does this work relate to a public health issue?**

- Metabolic syndrome is a major public health challenge associated with increased risk of cardiovascular disease, type 2 diabetes, and premature mortality.
- Community-based lifestyle interventions that combine exercise and health education represent accessible and potentially effective strategies for reducing cardiometabolic risk in adult populations.

**Public health significance—Why is this work of significance to public health?**

- This study provides a comparative reanalysis of a structured community-based intervention for adults with metabolic syndrome implemented in Chile.
- The findings indicate that participants who completed the intervention achieved significant improvements across multiple anthropometric, cardiometabolic, functional-capacity, and aerobic-fitness outcomes, with more favorable post-intervention values than adults who did not participate in the program.

**Public health implications—What are the key implications or messages for practitioners, policy makers and/or researchers in public health?**

- Integrated programs combining supervised aerobic exercise, health education, and clinical monitoring may support cardiometabolic risk reduction in community settings.
- The intervention model documented in this study may provide a feasible framework for adaptation within primary healthcare systems and public health promotion initiatives, particularly in Latin America and other resource-constrained settings.

**Abstract:**

Metabolic syndrome is a major public health concern, yet evidence from structured community-based interventions in Latin America remains limited. This study conducted a comparative reanalysis of an 18-week program implemented in Talca, Chile, that combined supervised aerobic exercise, health education, and clinical monitoring. The analysis included archived data from 80 adults with metabolic syndrome who had originally been randomly allocated using SPSS to an intervention group (n=33) or a usual-care comparison group (n=47). Anthropometric, biochemical, cardiovascular, body-composition, functional-capacity, and estimated cardiorespiratory-fitness outcomes were assessed at baseline and after 18 weeks. At the post-intervention assessment, the intervention group showed greater Six-Minute Walk Test (6MWT) distance, higher 6MWT-based estimates of cardiorespiratory fitness, lower waist circumference, systolic and diastolic blood pressure, fasting glucose, triglycerides, body weight, body fat percentage, and fat mass, and higher high-density lipoprotein (HDL) cholesterol and muscle mass percentage than the comparison group. Within the intervention group, body weight, body fat percentage, and fat mass decreased, whereas muscle mass and 6MWT distance increased. These findings indicate that participation in the integrated program was associated with favorable cardiometabolic, body-composition, and functional outcomes. The results support further prospective evaluation of this community-based model within primary healthcare and public health settings.

## 1. Introduction

Metabolic syndrome (MetS) is a multifactorial cardiometabolic disorder characterized by central obesity, elevated blood pressure, dyslipidemia, impaired glucose regulation, and insulin resistance [1,2,3]. Its increasing prevalence, driven partly by obesity and sedentary lifestyles, contributes substantially to cardiovascular disease, type 2 diabetes mellitus, premature mortality, and the growing burden of chronic disease on healthcare systems [4,5,6]. Consequently, prevention and integrated management of MetS remain priorities for clinical practice and public health [7].

**Figure 1:**
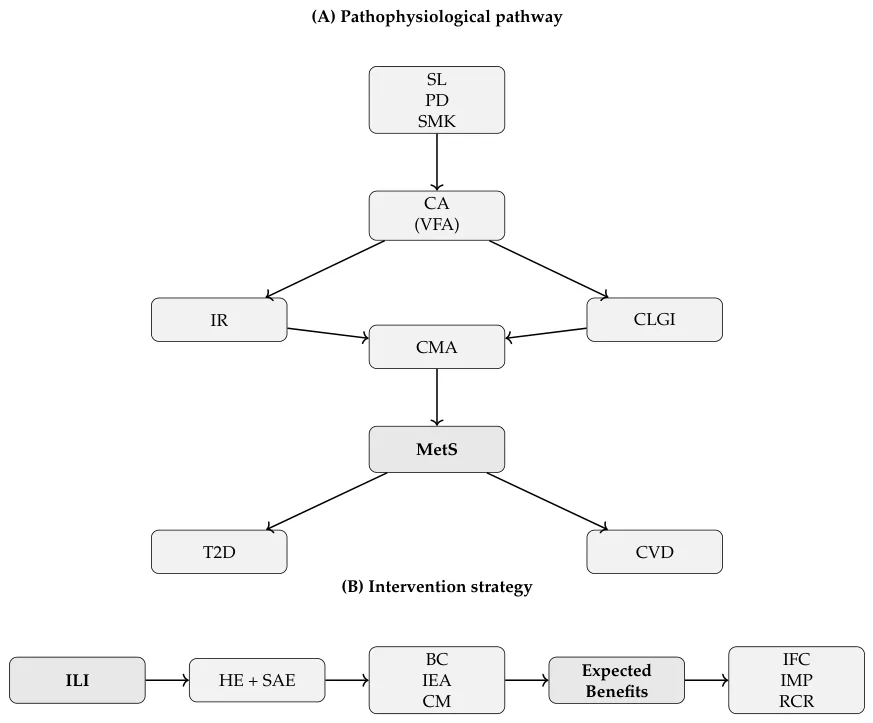
Conceptual framework of the study. Panel (**A**) illustrates the principal pathophysiological mechanisms associated with metabolic syndrome, whereas Panel (**B**) summarizes the integrated lifestyle intervention evaluated in the present investigation. *Abbreviations:* SL, sedentary lifestyle; PD, poor diet; SMK, smoking; CA, central adiposity; VFA, visceral fat accumulation; IR, insulin resistance; CLGI, chronic low-grade inflammation; CMA, cardiometabolic abnormalities; MetS, metabolic syndrome; T2D, type 2 diabetes; CVD, cardiovascular disease; ILI, integrated lifestyle intervention; HE, health education; SAE, supervised aerobic exercise; BC, behavioral change; IEA, improved exercise adherence; CM, cardiometabolic monitoring; IFC, improved functional capacity; IMP, improved metabolic profile; and RCR, reduced cardiometabolic risk.

Figure 1 summarizes the conceptual pathway underlying the study. Panel (A) shows how lifestyle-related exposures may promote central adiposity and visceral fat accumulation, contributing to insulin resistance, chronic low-grade inflammation, and cardiometabolic abnormalities. Panel (B) represents the intervention pathway, in which health education and supervised aerobic exercise support behavioral change, exercise adherence, and cardiometabolic monitoring, leading to improved functional capacity and metabolic profile and reduced cardiometabolic risk [8].

Lifestyle modification remains the cornerstone of metabolic syndrome management. Current guidelines recommend regular physical activity, healthy dietary habits, weight management, and behavioral support as first-line non-pharmacological strategies [9,10]. Regular aerobic exercise promotes favorable adaptations in glucose regulation, lipid metabolism, cardiovascular function, body composition, and cardiorespiratory fitness [11,12,13,14]. Health education complements these physiological effects by strengthening health literacy, self-management, adherence, and sustained behavioral change [15,16,17]. Interventions that combine exercise and educational support may therefore produce broader benefits than programs addressing either component in isolation [18,19].

Community-based multidisciplinary programs can translate these components into accessible interventions implemented through existing healthcare infrastructure, while also facilitating social support and participant engagement [20,21,22,23]. Nevertheless, substantial heterogeneity remains in intervention duration, exercise prescription, educational content, participant characteristics, and outcome assessment [24,25,26]. Evidence from Latin American populations is also comparatively limited, supporting the need to evaluate integrated programs under real-world community conditions.

The present investigation builds upon the community-based non-pharmacological intervention originally developed by Urzúa for adults with metabolic syndrome in Talca, Chile [27]. It addresses the limited availability of Latin American evidence and the scarcity of comparative evaluations conducted under community conditions. The novelty of the study lies in the comparative reanalysis of the original intervention and usual-care groups rather than in establishing the general benefits of exercise. Accordingly, the study aimed to compare anthropometric, cardiometabolic, body-composition, functional-capacity, and estimated cardiorespiratory-fitness outcomes between groups after 18 weeks, while also characterizing longitudinal changes within the intervention group.

## 2. Materials and Methods

### 2.1. Study Design and Participants

A secondary comparative reanalysis was conducted using archived data from a randomized community-based intervention program implemented in Talca, Chile. Participants were recruited from the same community cardiovascular-risk program and were considered eligible when they were 40–65 years of age, met the modified National Cholesterol Education Program Adult Treatment Panel III (NCEP ATP III) diagnostic criteria for metabolic syndrome, and had no medical condition that contraindicated moderate physical activity or prevented completion of the assessment procedures [28,29,30]. Eligible individuals received a detailed explanation of the study objectives, procedures, potential benefits, and risks and provided written informed consent before enrollment in accordance with internationally recognized ethical principles [31].

All eligible and consenting participants completed the same baseline clinical, anthropometric, biochemical, and functional assessments before group allocation. The archived doctoral records indicate that participants were subsequently randomly allocated using SPSS version 15.0 to the intervention group (n=33) or the usual-care comparison group (n=47) [27]. The available documentation does not indicate that allocation was performed sequentially according to recruitment order. However, it does not retain sufficient detail regarding the specific sequence-generation command, allocation ratio, allocation concealment, or personnel responsible for implementing the randomization procedure.

No individual matching or matched-pair allocation procedure was documented in the original records, and no post hoc matching was performed for the present reanalysis. Consequently, comparability was evaluated at the group level using the available baseline variables rather than through individually matched participants. These variables included age, sex distribution, body weight, height, BMI, 6MWT distance, cardiovascular responses, body fat percentage, fat mass, muscle mass, and the available sex-stratified blood pressure, fasting glucose, triglyceride, total cholesterol, and HDL-cholesterol measurements.

Most of the available demographic, anthropometric, functional, body-composition, and sex-stratified cardiometabolic variables did not differ significantly between groups at baseline. Nevertheless, the intervention group exhibited higher post-exercise systolic blood pressure and resting heart rate, and men in the intervention group exhibited higher median HDL cholesterol than men in the comparison group. Therefore, the groups were broadly comparable across several available characteristics but were not completely equivalent at baseline. These imbalances were considered when interpreting the post-intervention comparisons and are reported in Table 1.

#### Sample Size Considerations

The sample size for the present secondary reanalysis was determined by the number of eligible participants included in the archived original cohort. All 80 participants with available group-allocation information and baseline and post-intervention records were included: 33 in the intervention group and 47 in the usual-care comparison group. No additional participants were recruited, no observations were sampled from the archived cohort, and no retrospective exclusions were introduced to obtain balanced group sizes.

A formal a priori sample-size calculation for the original intervention was not retained in the archived documentation available for the present study. Consequently, no new prospective power calculation was applicable to this retrospective reanalysis. Including the complete available cohort maximized the use of the historical data and avoided further loss of statistical information. Nevertheless, the fixed and modest sample size, the unequal group sizes, and the limited availability of historical covariates reduce statistical precision and restrict the generalizability of the findings.

### 2.2. Scope of the Comparative Reanalysis

The participants, original group allocation, intervention protocol, assessment schedule, and archived clinical, anthropometric, biochemical, and functional records analyzed in this study originated from the doctoral project conducted by Urzúa in Talca, Chile [27]. No new participants were recruited, and no new biological samples or outcome measurements were collected for the present manuscript. The current study therefore constitutes a secondary comparative reanalysis of the complete available baseline and post-intervention records from the original intervention and usual-care comparison groups.

The present reanalysis focused on five components: (i) assessment of baseline comparability between the intervention and comparison groups; (ii) post-intervention comparisons between groups; (iii) baseline-to-post-intervention changes within the intervention group; (iv) calculation of relative between-group differences for selected functional and cardiometabolic outcomes; and (v) description of participant-level favorable responses when these records were available. Intermediate measurements and analyses not directly related to these objectives were outside the scope of the present article.

The scientific rationale for the reanalysis was to determine whether the outcomes observed among participants who completed the lifestyle program were more favorable than those observed among adults in the comparison group and to reinterpret the original community-based intervention using a contemporary public health reporting framework. The intervention protocol, group allocation, measurement procedures, and original data were not modified retrospectively; the added contribution of the present manuscript derives from the systematic comparative analysis and integrated interpretation of the archived outcomes.

### 2.3. Comparison Condition

Participants allocated to the comparison group continued receiving the usual clinical care available through the community cardiovascular-risk program during the same 18-week observation period. They completed the same baseline and post-intervention assessments as participants in the intervention group but did not attend the structured supervised-exercise or health-education sessions.

The absence of the structured intervention in this group was an intentional component of the original comparative design. The comparison condition was included to provide a concurrent reference for distinguishing changes associated with usual care, spontaneous behavioral modification, repeated assessment, regression to the mean, or other time-related factors from the changes observed among participants assigned to the integrated lifestyle program. The comparison group was therefore not excluded from treatment because of ineligibility or inability to exercise; rather, eligible participants had been assigned to continue usual care under the original study design.

The present reanalysis uses this usual-care group to contextualize the magnitude and direction of the post-intervention outcomes. Because complete information on possible behavioral changes, additional physical activity, dietary modification, medication changes, and healthcare use during follow-up was not retained, the comparison condition should be interpreted as usual care rather than as a no-contact or no-treatment control condition.

### 2.4. Integrated Lifestyle Intervention

The intervention consisted of an 18-week community-based lifestyle program integrating supervised aerobic exercise, structured health education, and clinical monitoring [27]. Participants attended three supervised exercise sessions per week, each lasting approximately 60 min. Each session included a warm-up, a controlled-intensity aerobic-exercise phase, and a cool-down. Exercise intensity and progression were individualized according to physical capacity, cardiovascular response, exercise tolerance, and clinical judgment.

Structured health-education activities were delivered weekly. The educational content addressed metabolic syndrome, cardiovascular-disease prevention, healthy eating, regular physical activity, weight management, smoking cessation, responsible alcohol consumption, and strategies for sustaining healthier behaviors. Clinical monitoring continued throughout the intervention period to support participant safety and guide individualized exercise progression.

The integrated structure was intended to address complementary physiological and behavioral determinants of cardiometabolic risk. Similar programs combining supervised physical activity and educational support have been associated with favorable cardiometabolic, functional, and self-management outcomes [18,19,20,32].

### 2.5. Outcome Measures and Assessment Procedures

Baseline and post-intervention assessments were conducted using the same standardized clinical, anthropometric, biochemical, and functional procedures. The assessment protocol included body size and composition, cardiovascular responses, fasting biochemical markers, functional walking capacity, and estimated cardiorespiratory fitness. The original measurements were conducted by trained personnel associated with the community cardiovascular-risk program, following the procedures documented in the doctoral project [27].

Anthropometric assessments included body weight, height, body mass index (BMI), waist circumference, hip circumference, waist-to-hip ratio, body fat percentage, fat mass, and muscle mass. Participants were evaluated wearing light clothing and without shoes. Body weight was measured using a calibrated clinical scale, height was measured using a wall-mounted stadiometer, and BMI was calculated as body weight divided by height squared (kg/m^2^). Waist and hip circumferences were obtained using a non-elastic anthropometric tape according to standardized anatomical landmarks.

Body fat percentage, fat mass, and muscle mass were estimated using the standardized anthropometric procedure described in the original doctoral protocol, which incorporated skinfold, circumference, and body-dimension measurements [27]. Anatomical landmarks were identified before measurement, and the instruments were checked and calibrated before each assessment. All evaluations were performed by trained assessors using the same procedures at baseline and after completion of the 18-week program. The manufacturers and models of the clinical scale, stadiometer, anthropometric tape, and skinfold caliper were not retained in the archived documentation.

#### 2.5.1. Cardiovascular Measurements

Cardiovascular measurements included resting and post-exercise heart rate, systolic blood pressure, diastolic blood pressure, and peripheral oxygen saturation. Before functional testing, participants remained seated at rest for 10–15 min to allow cardiovascular parameters to stabilize. Blood pressure was measured manually using a sphygmomanometer and a Littmann stethoscope (3M Health Care, St. Paul, MN, USA), heart rate was recorded using Polar heart-rate monitors (Polar Electro Oy, Kempele, Finland), and peripheral oxygen saturation was assessed using a Model 8600 pulse oximeter (Nonin Medical, Inc., Plymouth, MN, USA). The manufacturer and model of the sphygmomanometer were not retained in the archived documentation.

The archived protocol documented one standardized blood pressure measurement before testing and one measurement immediately after completion of each functional assessment rather than duplicate or triplicate resting measurements. Therefore, the reported pre-test and post-test values correspond to single standardized assessments obtained under the same procedural conditions. Cardiovascular measurements and participant symptoms were monitored by trained healthcare personnel throughout the functional tests.

#### 2.5.2. Biochemical Analyses

Venous blood samples were collected following a 12-h overnight fast. The biochemical assessment included fasting glucose, total cholesterol, high-density lipoprotein (HDL) cholesterol, low-density lipoprotein (LDL) cholesterol, and triglycerides. Samples collected at baseline and follow-up were analyzed under the same laboratory conditions using specific reagent kits for each determination (Roche Laboratories, Mannheim, Germany) and an automated Hitachi 717 spectrophotometer (Hitachi, Ltd., Tokyo, Japan). LDL cholesterol was calculated using the Friedewald equation when the analytical conditions required for its application were satisfied [27].

Blood collection and laboratory processing were conducted by qualified clinical laboratory personnel associated with the cardiovascular-risk research program. The archived documentation did not retain the names or individual professional categories of the personnel who performed each venipuncture; consequently, the procedure is reported at the level supported by the historical records. The same preanalytical fasting requirements and analytical procedures were applied at each assessment point.

#### 2.5.3. Six-Minute Walk Test

Functional capacity was assessed using the Six-Minute Walk Test (6MWT). The test was conducted on a flat, level, unobstructed walking course of approximately 30 m, with turning points identified using floor markings and signaling cones. Participants wore comfortable clothing and walking shoes, avoided food intake during the two hours preceding the assessment, and rested for 10–15 min before starting the test.

Participants were instructed to walk, without running, as far as possible during six minutes at a self-selected pace. Total walking distance was recorded in meters as the principal functional outcome. The equipment used included a Roto-Sure 1000 Range distance-measuring wheel (Roto-Plastics (Pty) Ltd., Boksburg, South Africa), four digital stopwatches, signaling cones, a manual sphygmomanometer, a Littmann stethoscope (3M Health Care, St. Paul, MN, USA), and a Model 8600 pulse oximeter (Nonin Medical, Inc., Plymouth, MN, USA). Heart rate, blood pressure, and peripheral oxygen saturation were recorded before and immediately after the test [27]. The manufacturers and models of the stopwatches, signaling cones, and manual sphygmomanometer were not retained in the archived documentation.

#### 2.5.4. Bruce Treadmill Protocol

Estimated cardiorespiratory fitness was assessed using the graded Bruce treadmill protocol on a Technogym treadmill. The test consisted of successive three-minute stages with progressive increases in treadmill speed and inclination. Participants continued until volitional exhaustion, achievement of the required exercise intensity, or termination by the supervising healthcare professional because of symptoms or an abnormal clinical response. A test reaching at least 85% of the age-predicted maximum heart rate was considered physiologically conclusive according to the original protocol.

Heart rate was monitored using Polar devices, while blood pressure, peripheral oxygen saturation, and participant symptoms were assessed throughout testing using the clinical equipment described above. Direct respiratory gas analysis was not performed. Consequently, oxygen-consumption outcomes were interpreted as treadmill-derived estimates rather than direct measurements of maximal oxygen uptake.

Estimated relative ${\hat{\mathrm{VO}}}_{2\mathrm{peak}}$ was calculated from total Bruce treadmill duration using the original sex-specific equations:

(1) $${\hat{\mathrm{VO}}}_{2\mathrm{peak}}=8.33+2.94\;T,\;\mathrm{for}\;\mathrm{men},$$

(2) $${\hat{\mathrm{VO}}}_{2\mathrm{peak}}=8.05+2.74\;T,\;\mathrm{for}\;\mathrm{women},$$

where *T* represents total treadmill duration in minutes, and ${\hat{\mathrm{VO}}}_{2\mathrm{peak}}$ is expressed in $\mathrm{mL}\cdot {\mathrm{kg}}^{-1}\cdot \min^{-1}$ [27].

#### 2.5.5. Six-Minute Walk Test-Based Prediction Models

In addition to the treadmill-derived estimate obtained from the Bruce protocol, two multivariable prediction equations based on Six-Minute Walk Test performance were applied as complementary indicators of cardiorespiratory fitness. These models incorporated walking distance and selected demographic, anthropometric, and cardiovascular variables and therefore represent indirect estimates rather than oxygen-consumption measurements obtained through respiratory gas analysis [27].

Model 1 estimated relative VO_2_ peak using the following equation:

(3) $${\hat{\mathrm{VO}}}_{2\mathrm{peak}}=0.02D-0.191A-0.07W+0.09H+0.26RPP+2.45,$$

where *D* represents the 6MWT distance in meters, *A* is age in years, *W* is body weight in kilograms, *H* is height in centimeters, and RPP is the rate–pressure product calculated as systolic blood pressure in mmHg multiplied by heart rate in beats per minute and expressed on a $10^{-3}$ scale. The output of Model 1 is expressed in mL·kg^−1^·min^−1^.

Model 2 estimated percent-predicted VO_2_ peak using the following equation:

(4) $${\hat{\mathrm{VO}}}_{2\mathrm{peak}}\left(\%\right)=0.05D-0.22A+0.27W+0.14H+0.78RPP+26.16,$$

The variables included in Model 2 were defined in the same manner as in Model 1. However, the output of Model 2 is expressed as percent-predicted VO_2_ peak rather than in mL·kg^−1^·min^−1^. Accordingly, the value of 102.0 reported for the intervention group represents 102.0% of the predicted VO_2_ peak and must not be interpreted as an oxygen-consumption value of 102.0 mL·kg^−1^·min^−1^. Both equation-derived estimates are distinct from the VO_2_ peak estimate obtained from total treadmill duration during the Bruce protocol, and none represents oxygen uptake measured directly through respiratory gas analysis.

### 2.6. Statistical Analysis

Statistical analyses were performed using SPSS version 15.0. Continuous variables were summarized as mean and standard deviation when the corresponding parametric assumptions were satisfied and as median and interquartile range when these assumptions were not met. Categorical variables were summarized as frequencies and percentages. Normality was evaluated using the Kolmogorov–Smirnov test, and homogeneity of variances was assessed using Levene’s test.

Baseline and post-intervention comparisons between the intervention and usual-care comparison groups were conducted using the independent-samples Student’s *t*-test for normally distributed continuous variables and the Mann–Whitney *U* test for continuous variables that did not satisfy parametric assumptions. Categorical baseline variables, including sex distribution, were compared using the chi-square test or Fisher’s exact test when expected cell frequencies were insufficient for the chi-square approximation.

Baseline-to-post-intervention changes within the intervention group were evaluated using the paired-samples Student’s *t*-test for normally distributed paired differences and the Wilcoxon signed-rank test when the distributional assumption was not satisfied. All statistical tests were two-sided. Statistical significance was established at p<0.05, and the reported *p*-values correspond to the specific comparisons identified in the tables and accompanying text. No multivariable adjustment was performed because several potentially relevant historical covariates were not consistently retained in the archived dataset.

For selected post-intervention outcomes, relative between-group differences were calculated using the comparison-group mean as the reference value:

(5) $$\mathrm{Relative}\;\mathrm{difference}\;\left(\%\right)=100\times \frac{{\bar{X}}_{I}-{\bar{X}}_{C}}{{\bar{X}}_{C}},$$

where ${\bar{X}}_{I}$ and ${\bar{X}}_{C}$ represent the post-intervention means of the intervention and comparison groups, respectively. Under this calculation, positive values indicate that the intervention-group mean was higher than the comparison-group mean, whereas negative values indicate that it was lower. The clinical interpretation depends on the direction of benefit for each outcome: a positive value favors the intervention group for functional outcomes such as 6MWT distance, whereas a negative value favors the intervention group for cardiometabolic risk indicators such as waist circumference, systolic blood pressure, fasting glucose, and triglycerides.

## 3. Results

A total of 80 adults diagnosed with metabolic syndrome were included in the comparative reanalysis. In the original study, participants were randomly allocated using SPSS version 15.0 to the intervention group (n=33) or the comparison group (n=47). Baseline and post-intervention assessments were available for both groups, enabling the evaluation of within-group changes over time and comparative analyses between the randomized groups.

### 3.1. Baseline Characteristics

Table 1 presents the available baseline demographic, anthropometric, cardiovascular, body-composition, biochemical, and functional characteristics of the intervention and comparison groups. The study sample included 47 women and 33 men. The comparison group comprised 24 women and 23 men, whereas the intervention group comprised 23 women and 10 men. Most demographic, anthropometric, functional, and body-composition variables did not differ significantly between groups at baseline. However, selected cardiovascular and sex-stratified cardiometabolic differences were identified and were considered when interpreting the post-intervention comparisons.

No statistically significant baseline differences were observed for age, sex distribution, body weight, height, body mass index, walking distance, body fat, fat mass, or muscle mass. However, post-exercise systolic blood pressure was higher in the intervention group than in the comparison group (151.1±22.2 vs. 135.7±16.0 mmHg, p=0.001), and resting heart rate was also higher in the intervention group (79.2±11.5 vs. 73.8±8.5 bpm, p=0.025). In the sex-stratified analysis, median HDL cholesterol was higher among men assigned to the intervention group than among men assigned to the comparison group (41.0 [IQR 10.0] vs. 34.5 [IQR 7.0] mg/dL, p=0.040). These findings indicate that random allocation did not produce complete baseline equivalence across all available variables.

Group-level baseline summaries for waist-to-hip ratio, habitual physical activity, smoking status, and concurrent pharmacotherapy were not systematically retained in the archived analytical dataset and therefore could not be incorporated into Table 1. This limitation was considered when interpreting the between-group findings.

### 3.2. Between-Group Post-Intervention Comparisons

**Figure 2:**
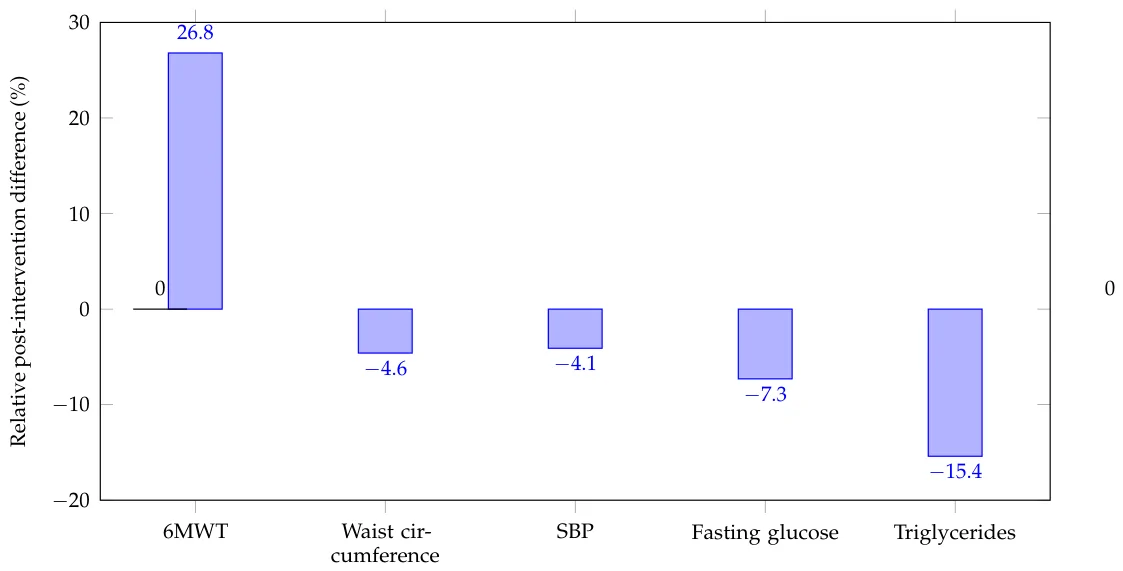
Relative post-intervention differences between the intervention and comparison groups for selected functional and cardiometabolic outcomes. Relative differences were calculated as $100\times \left({\bar{X}}_{I}-{\bar{X}}_{C}\right)/{\bar{X}}_{C}$, where ${\bar{X}}_{I}$ and ${\bar{X}}_{C}$ denote the intervention- and comparison-group post-intervention means, respectively. The plotted values were 26.8% for 6MWT distance (p<0.001), −4.6% for waist circumference (p=0.018), −4.1% for systolic blood pressure (p=0.041), −7.3% for fasting glucose (p=0.027), and −15.4% for triglycerides (p=0.009). A positive value for 6MWT distance indicates higher functional performance in the intervention group, whereas negative values for the cardiometabolic indicators indicate lower and therefore more favorable values in the intervention group. Absolute group means, standard deviations, measurement units, and between-group *p*-values are reported in Table 2. *Abbreviations:* 6MWT, Six-Minute Walk Test; SBP, systolic blood pressure.

Post-intervention comparisons between the intervention and comparison groups are presented in Table 2. Relative between-group differences for selected statistically significant functional and cardiometabolic outcomes are summarized in Figure 2.

The post-intervention comparison showed statistically significant differences favoring the intervention group across functional-capacity, estimated aerobic-fitness, cardiovascular, biochemical, anthropometric, and body-composition outcomes. Participants in the intervention group exhibited greater 6MWT distance, greater predicted 6MWT distance, and higher 6MWT-based estimated cardiorespiratory-fitness values than participants in the comparison group. They also exhibited lower waist circumference, systolic and diastolic blood pressure, fasting glucose, triglycerides, body weight, body fat percentage, and fat mass and higher HDL cholesterol and muscle mass percentage. By contrast, absolute muscle mass and BMI did not differ significantly between groups.

Among the body-composition outcomes, post-intervention fat mass was 23.1±5.1 kg in the intervention group and 34.1±12.0 kg in the comparison group (p<0.001). Post-intervention body fat percentage was also lower in the intervention group (29.8±4.3% vs. 33.7±6.3%, p=0.003), whereas muscle mass percentage was higher (40.9±3.5% vs. 37.2±4.6%, p=0.001). Absolute muscle mass was nearly identical between groups (31.7±5.3 vs. 31.6±7.1 kg, p=0.931). Therefore, the post-intervention comparison supports lower adiposity and a higher relative contribution of muscle mass in the intervention group, but it does not demonstrate a significant between-group difference in absolute muscle mass.

Figure 2 complements the absolute post-intervention comparisons reported in Table 2 by presenting the relative differences calculated according to Equation (5). The figure includes one functional outcome, 6MWT distance, and four cardiometabolic risk indicators: waist circumference, systolic blood pressure, fasting glucose, and triglycerides. Accordingly, a positive relative difference for 6MWT distance indicates greater functional performance in the intervention group, whereas negative relative differences for the four cardiometabolic indicators indicate lower and therefore more favorable post-intervention values in the intervention group.

### 3.3. Within-Group Changes in the Intervention Group

**Figure 3:**
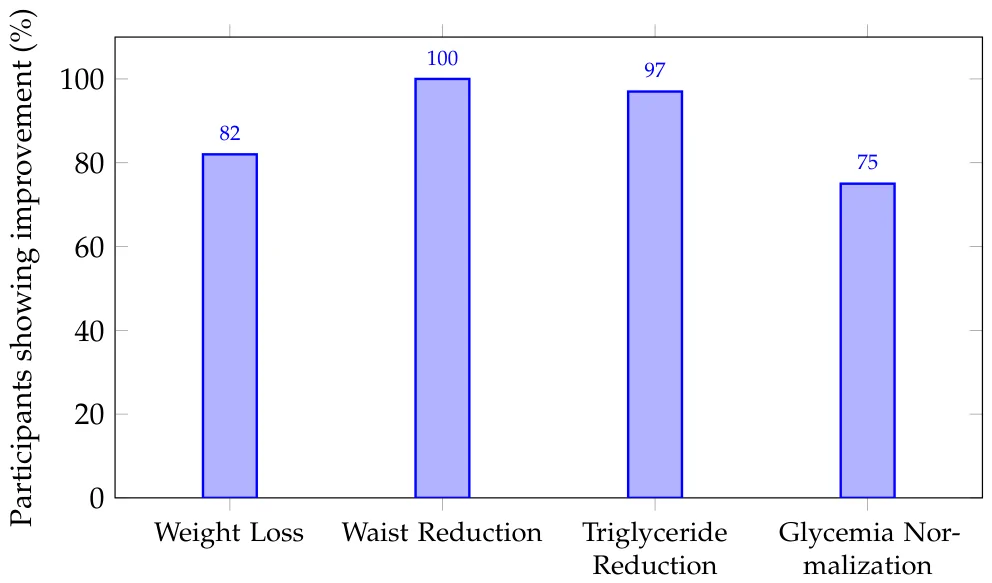
Percentage of intervention participants showing favorable changes in selected anthropometric and metabolic outcomes after completion of the 18-week integrated lifestyle intervention. The percentage for fasting glucose normalization was calculated among participants with abnormal fasting glucose at baseline.

Baseline-to-post-intervention changes within the intervention group are presented in Table 3. Participant-level favorable responses for selected anthropometric and metabolic outcomes are summarized in Figure 3. Table 3 describes changes in group-level means, whereas Figure 3 presents the proportions of participants who experienced favorable individual responses.

Within the intervention group, body weight decreased from 80.9±11.5 to 77.2±10.9 kg, corresponding to a relative reduction of 4.6%. Body fat percentage decreased from 35.1±5.4% to 29.8±4.3%, while fat mass decreased from 28.2±6.0 to 23.1±5.1 kg. These changes corresponded to relative reductions of 15.1% and 18.1%, respectively.

Muscle mass showed a more modest favorable longitudinal change. Muscle mass expressed as a percentage increased from 36.9±3.9% to 40.9±3.5%, while muscle mass expressed in kilograms increased from 29.8±5.5 to 31.7±5.3 kg. Functional capacity exhibited the largest relative improvement, with 6MWT distance increasing from 523.8±90.8 to 641.0±79.8 m, corresponding to a 22.4% increase.

At the participant level, waist circumference decreased in all participants, while 27 participants experienced body weight loss. Figure 3 shows that favorable responses were observed for body weight in 82% of participants, waist circumference in 100%, and triglycerides in 97%. Among participants with abnormal fasting glucose at baseline, 75% achieved normalization by the end of the intervention.

The universal reduction in waist circumference is particularly relevant because central adiposity is a defining component of metabolic syndrome and is strongly associated with insulin resistance and elevated cardiometabolic risk. The participant-level responses shown in Figure 3 complement the changes in group-level means reported in Table 3 by showing that favorable responses occurred across a substantial proportion of the intervention group.

## 4. Discussion

The present study comparatively reanalyzed archived data from an 18-week community-based lifestyle program for adults with metabolic syndrome. The original participants were allocated to an intervention group or a usual-care comparison group, and the present analysis jointly examined baseline comparability, post-intervention differences, longitudinal changes within the intervention group, and selected participant-level responses.

The overall pattern of findings was favorable to the intervention group across body-composition, cardiovascular, biochemical, functional-capacity, and estimated cardiorespiratory-fitness outcomes. The between-group differences were not limited to 6MWT performance and equation-derived fitness estimates; they also included waist circumference, blood pressure, fasting glucose, triglycerides, HDL cholesterol, body weight, body fat percentage, fat mass, and muscle mass percentage. Absolute muscle mass and BMI did not differ significantly between groups at the post-intervention assessment.

Although the benefits of exercise and lifestyle modification for metabolic syndrome are well established, the present study contributes implementation-oriented evidence from a Latin American community setting. The findings should be interpreted as associations observed in a retrospective reanalysis of archived data rather than as definitive evidence of causal effectiveness, long-term sustainability, or population-level scalability.

### 4.1. Body Composition and Central Obesity

The reduction in waist circumference observed among all participants in the intervention group represents one of the most consistent participant-level findings. Abdominal adiposity is widely recognized as a defining component of metabolic syndrome and is strongly associated with insulin resistance, chronic low-grade inflammation, and increased cardiovascular risk [1,8]. Consequently, changes in waist circumference may provide a more meaningful representation of metabolic improvement than body weight alone.

Although average body weight reduction was moderate, the intervention group exhibited substantial reductions in body fat percentage and fat mass. Body fat percentage decreased from 35.1±5.4% to 29.8±4.3%, while fat mass decreased from 28.2±6.0 to 23.1±5.1 kg. Muscle mass increased modestly within the intervention group, from 29.8±5.5 to 31.7±5.3 kg. Nevertheless, absolute muscle mass did not differ significantly between the intervention and comparison groups at the post-intervention assessment (31.7±5.3 vs. 31.6±7.1 kg, p=0.931). These findings indicate that the principal body-composition adaptations involved reductions in adiposity accompanied by a modest longitudinal increase in muscle mass, rather than a clear post-intervention difference in muscle mass between groups.

The combination of reduced abdominal adiposity, lower body fat percentage, and lower post-intervention fat mass may represent an important mechanism underlying the broader cardiometabolic benefits observed throughout the study. Although preservation of skeletal muscle is metabolically relevant because of its role in glucose uptake and insulin sensitivity, the present analysis did not demonstrate a statistically significant between-group difference in muscle mass. Previous investigations have shown that reductions in visceral adiposity, particularly when lean mass is preserved, are associated with improved metabolic health and lower cardiovascular risk [12].

### 4.2. Metabolic Improvements

The post-intervention cardiometabolic comparisons are presented in Table 2, whereas the participant-level fasting-glucose response is summarized in Figure 3. As shown in Table 2, fasting glucose, triglycerides, systolic blood pressure, and diastolic blood pressure were lower in the intervention group than in the comparison group, whereas HDL cholesterol was higher. These findings are consistent with physiological adaptations commonly associated with regular aerobic exercise, including improved lipid metabolism, endothelial function, vascular regulation, and glucose utilization [11,32].

At the participant level, Figure 3 shows that 75% of those with abnormal fasting glucose at baseline achieved normalization by the end of the intervention. This result suggests that the program may have been particularly relevant for participants presenting greater glycemic impairment at baseline. Nevertheless, because the present reanalysis did not include an adjusted longitudinal model for fasting glucose, this participant-level finding should be interpreted descriptively rather than as definitive evidence of an intervention effect.

Overall, the metabolic findings are consistent with the established role of lifestyle modification in cardiometabolic-risk management. However, the retrospective nature of the reanalysis, the modest sample size, and the incomplete availability of potential confounding variables limit causal interpretation [1,8,14].

### 4.3. Functional Capacity and Estimated Cardiorespiratory Fitness

Functional performance was more favorable in the intervention group at the post-intervention assessment. Participants in the intervention group walked a greater distance during the Six-Minute Walk Test and presented higher values for the two 6MWT-based cardiorespiratory-fitness estimates than participants in the comparison group. Within the intervention group, 6MWT distance increased from 523.8±90.8 to 641.0±79.8 m, corresponding to a relative increase of 22.4%.

Cardiorespiratory fitness is an important predictor of cardiovascular and all-cause mortality [5,12]. Nevertheless, the fitness indicators used in the present study were derived from functional-test performance and prediction equations rather than measured directly through respiratory gas analysis. Therefore, the observed differences should be interpreted as improvements in functional capacity and estimated cardiorespiratory fitness, not as directly measured changes in maximal oxygen uptake.

The concurrent findings for 6MWT performance, equation-derived fitness estimates, body composition, and cardiometabolic risk indicators indicate a consistent pattern of favorable outcomes across several physiological domains. However, the present results do not allow the separate contributions of supervised exercise, health education, clinical monitoring, or other unmeasured behavioral changes to be determined [33,34,35].

### 4.4. Practical and Public Health Implications

The findings support the practical relevance of integrated community-based lifestyle programs for adults with metabolic syndrome. The present program was implemented under community conditions and was associated with favorable differences across several clinically relevant anthropometric, cardiometabolic, body-composition, and functional outcomes [19,20,22].

The educational component may complement supervised exercise by supporting health literacy, self-management, and engagement with healthier behaviors [16,17,36,37]. Nevertheless, the present study did not independently evaluate behavioral adherence, dietary change, health literacy, or the specific contribution of each intervention component. Consequently, the mechanisms underlying the observed differences cannot be isolated from the available data, and the sustainability of these outcomes beyond the intervention period remains unknown.

From a public health perspective, the intervention represents a potentially adaptable model for primary healthcare and local health-promotion initiatives. However, feasibility across other populations and settings, long-term sustainability, resource requirements, and cost-effectiveness require direct evaluation before broader implementation can be recommended [21,23].

### 4.5. Strengths and Limitations

Several limitations should be considered. The present manuscript is based on a retrospective secondary reanalysis of archived data, and the sample size was fixed by the 80 participants available from the original cohort. A formal a priori sample-size calculation was not retained in the archived documentation, and the unequal numbers in the intervention and comparison groups may reduce statistical precision. The available records did not permit adjustment for all potentially relevant baseline characteristics, including habitual physical activity, dietary intake, smoking status, concurrent pharmacotherapy, socioeconomic conditions, and associated comorbidities. Some baseline imbalances remained despite the original random allocation, and no adjusted longitudinal model was available to determine the extent to which these differences influenced the post-intervention comparisons. The archived documentation also did not retain sufficient detail regarding sequence generation, allocation concealment, or implementation of the original randomization procedure.

The study did not include long-term follow-up, adherence analysis, intervention-fidelity assessment, or economic evaluation. Cardiorespiratory fitness was estimated from functional-test performance and prediction equations rather than measured directly through respiratory gas analysis. In addition, complete manufacturer, model, and calibration records were unavailable for some historical anthropometric and clinical instruments. These limitations restrict causal interpretation, procedural reproducibility, and conclusions regarding long-term sustainability, scalability, and cost-effectiveness.

## 5. Conclusions

This study provides a comparative reanalysis of an 18-week community-based lifestyle intervention for adults with metabolic syndrome in Talca, Chile. At the post-intervention assessment, the intervention group showed more favorable functional-capacity, estimated cardiorespiratory-fitness, anthropometric, body-composition, cardiovascular, and biochemical outcomes than the usual-care comparison group. Longitudinally, the intervention group showed reductions in body weight, body fat percentage, and fat mass, together with increases in muscle mass and 6MWT distance.

The main contribution of the study lies in documenting and comparatively reanalyzing a structured Latin American community-based intervention using archived data from the original intervention and comparison groups. The findings support the potential relevance of this integrated model within community and primary-healthcare settings. However, they should be interpreted as favorable associations identified through a retrospective secondary reanalysis rather than as definitive evidence of causal effectiveness, long-term sustainability, scalability, or cost-effectiveness.

Future research should use prospectively registered protocols, fully documented allocation and assessment procedures, larger samples, adjusted longitudinal analyses, direct measurement of cardiorespiratory fitness through respiratory gas analysis, long-term follow-up, adherence and intervention-fidelity assessment, and formal economic evaluation. Such studies are necessary to determine the reproducibility, sustainability, and broader public health applicability of this intervention model.

## Figures and Tables

**Table 1:** Baseline demographic, anthropometric, cardiovascular, body-composition, biochemical, and functional characteristics of the intervention and comparison groups.

**Panel A. Baseline characteristics available for the complete study groups**						
**Variable**		**Comparison Group** (n=47)		**Intervention Group** (n=33)		**p-Value**
Age (years)		52.0±5.6		51.1±6.4		0.504
Sex, female/male, *n*		24/23		23/10		0.096
Body weight (kg)		83.3±15.4		80.9±11.5		0.458
Height (cm)		162.0±9.9		160.0±9.6		0.370
Body mass index (kg/m^2^)		31.7±5.0		31.6±3.9		0.933
6MWT distance (m)		498.1±64.8		523.8±90.8		0.144
Post-exercise systolic blood pressure (mmHg)		135.7±16.0		151.1±22.2		**0.001**
Resting heart rate (bpm)		73.8±8.5		79.2±11.5		**0.025**
Post-exercise heart rate (bpm)		98.7±18.4		103.3±24.3		0.337
Body fat (%)		32.9±6.5		35.1±5.4		0.105
Fat mass (kg)		27.4±7.2		28.2±6.0		0.579
Muscle mass (%)		38.1±4.9		36.9±3.9		0.227
Muscle mass (kg)		32.0±7.6		29.8±5.5		0.151
**Panel B. Sex-stratified baseline cardiometabolic characteristics available in the archived records**						
**Variable**	**Women**			**Men**		
	**Comparison** (n=24)	**Intervention** (n=23)	** p **	**Comparison** (n=23)	**Intervention** (n=10)	** p **
Age (years)	51.3±4.2	48.7±4.8	0.150	50.3±6.9	53.3±6.6	0.370
Body weight (kg)	73.0±9.6	78.1±11.9	0.260	80.1±9.7	87.5±8.7	0.150
Waist circumference (cm)	101.8±10.6	107.2±10.4	0.180	99.3±7.6	101.5±8.2	0.540
Body mass index (kg/m^2^)	30.9±3.6	33.0±4.1	0.310	27.1±2.8	29.4±2.5	0.080
Systolic blood pressure (mmHg)	137.6±13.4	133.8±15.0	0.490	138.5±17.1	138.4±6.7	0.990
Diastolic blood pressure (mmHg)	83.2±12.5	83.9±11.2	0.870	82.3±12.7	88.3±8.6	0.230
Triglycerides (mg/dL)	162.0(97.0)	198.0(63.0)	0.080	185.5(243.0)	166.0(141.0)	0.890
Total cholesterol (mg/dL)	216.9±24.4	203.6±31.3	0.220	173.2±29.4	188.4±33.8	0.280
HDL cholesterol (mg/dL)	41.5(15.0)	45.5(10.0)	0.340	34.5(7.0)	41.0(10.0)	**0.040**
Fasting glucose (mg/dL)	99.1±11.3	99.3±10.5	0.950	100.5±8.3	99.4±9.2	0.790

Note: Values are reported as mean ± standard deviation, except triglycerides and HDL cholesterol, which are reported as median (interquartile range). Continuous normally distributed variables were compared using independent-samples Student’s *t*-tests, whereas triglycerides and HDL cholesterol were compared using the Mann–Whitney *U* test. Sex distribution was compared using the chi-square test. Statistical significance was established at p<0.05. 6MWT, Six-Minute Walk Test; HDL, high-density lipoprotein.

**Table 2:** Post-intervention comparison between the intervention and comparison groups.

Variable	Comparison Group		Intervention Group		*p*-Value
	Mean	SD	Mean	SD	
6MWT distance (m)	505.7	57.6	641.0	79.8	<0.001
Predicted 6MWT distance (m)	507.5	40.4	538.6	51.9	0.004
6MWT-based estimated VO_2_ peak, Model 1 (mL·kg^−1^·min^−1^)	22.9	2.6	24.2	2.1	0.020
6MWT-based percent-predicted VO_2_ peak, Model 2 (%)	65.0	5.6	102.0	6.7	<0.001
Waist circumference (cm)	105.8	9.4	100.9	8.8	0.018
Systolic blood pressure (mmHg)	138.4	12.7	132.7	11.9	0.041
Diastolic blood pressure (mmHg)	86.9	8.1	81.6	7.5	0.012
Fasting glucose (mg/dL)	111.8	13.8	103.6	12.1	0.027
Triglycerides (mg/dL)	194.2	52.6	164.2	45.3	0.009
HDL cholesterol (mg/dL)	41.3	6.8	45.1	7.4	0.036
Body weight (kg)	84.7	14.5	77.2	10.9	0.018
Body fat (%)	33.7	6.3	29.8	4.3	0.003
Fat mass (kg)	34.1	12.0	23.1	5.1	<0.001
Muscle mass (%)	37.2	4.6	40.9	3.5	0.001
Muscle mass (kg)	31.6	7.1	31.7	5.3	0.931
BMI (kg/m^2^)	32.3	4.8	30.9	3.9	0.190

Note: Data are reported as mean and standard deviation (SD). *p*-values represent post-intervention comparisons between the intervention and comparison groups and were calculated using the independent-samples Student’s *t*-test or Mann–Whitney *U* test, as appropriate. Statistical significance was established at p<0.05. Model 1 is expressed in mL·kg^−1^·min^−1^, whereas Model 2 is expressed as percent-predicted VO_2_ peak. Thus, the value 102.0 reported for Model 2 represents 102.0% of the predicted value and not 102.0 mL·kg^−1^·min^−1^. 6MWT, Six-Minute Walk Test; HDL, high-density lipoprotein; BMI, body mass index; and RPP, rate–pressure product.

**Table 3:** Baseline-to-post-intervention changes within the intervention group.

Variable	Baseline	Post-Intervention	% Change	*p*-Value
Body weight (kg)	80.9±11.5	77.2±10.9	−4.6	<0.05
Body fat (%)	35.1±5.4	29.8±4.3	−15.1	<0.05
Fat mass (kg)	28.2±6.0	23.1±5.1	−18.1	<0.05
Muscle mass (%)	36.9±3.9	40.9±3.5	+10.8	<0.05
Muscle mass (kg)	29.8±5.5	31.7±5.3	+6.4	<0.05
6MWT distance (m)	523.8±90.8	641.0±79.8	+22.4	<0.001

Note: Values are reported as mean ± standard deviation. Percentage change was calculated as 100 × (post-intervention value − baseline value)/baseline value. The baseline values correspond to the intervention group reported in Table 1, whereas the post-intervention values correspond to the same intervention group reported in Table 2. Baseline-to-post-intervention differences were evaluated using the paired-samples Student’s *t*-test or Wilcoxon signed-rank test, as appropriate. Statistical significance was established at p<0.05. 6MWT, Six-Minute Walk Test.

## Data Availability

The original contributions presented in this study are included in the article/Supplementary Material. Further inquiries can be directed to the corresponding authors.

## Supplementary Materials

The following supporting information can be downloaded at: https://github.com/cvidalmsu/tesis-doctorales-urzua-parada (accessed on 6 June 2026), Supplementary materials include the variable dictionary, metadata specification, repository documentation, example graph representations, and computational resources distributed through the public GitHub repository associated with this dataset.
